# Efficacy of Plasmalogens on Monosodium Glutamate-Induced Neurotoxicity in Male Rats Through NF-*κ*B and p38 MAPK Signaling Pathways

**DOI:** 10.1155/omcl/3673280

**Published:** 2025-04-04

**Authors:** Heba M. Abdou, Fatma A. Hamaad, Ghada M. Abd Elmageed, Hideki Katano, Mamdooh H. Ghoneum

**Affiliations:** ^1^Department of Zoology, Alexandria University, Alexandria, Egypt; ^2^Department of Biochemistry, Alexandria University, Alexandria, Egypt; ^3^Institute of Gerontology, Adachi-ku, Tokyo, Japan; ^4^Department of Surgery, Charles R. Drew University of Medicine and Science, Los Angeles, California, USA; ^5^Department of Surgery, University of California Los Angeles, Los Angeles, California, USA

**Keywords:** monosodium glutamate, neurochemical markers, neurodegeneration, p38 MAPK, plasmalogens

## Abstract

Monosodium glutamate (MSG) is the most commonly used food additive and has well-known neurotoxic effects. The current study was carried out to assess the underlying mechanisms of the neurotoxicity of MSG on the hippocampus in male rats and examine the protective effect of plasmalogens (Pls) on nuclear factor-B (NF-*κ*B) and p38 MAPK signaling pathways in the hippocampus using behavioral, biochemical, and immunohistochemical methods. Twenty-four male Wistar albino rats were divided into four groups for control or treatment with MSG (2 g/kg body weight) and/or Pls (100 mg/kg body weight). All doses were received orally for 28 days. Results show that plasmalogens ameliorate the levels of glucose, insulin, lipids, oxidative stress markers, antioxidant enzymes, AKT, and neurochemical markers. It also reduces the level of the inflammatory markers TNF-*α*, NF-*κ*B, and p38 mitogen-activated protein kinase (MAPK). Histological and immunohistochemical alterations in hippocampal tissues were found to be augmented postexposure to Pls, suggesting that Pls have a potent ameliorative effect. We conclude that Pls exert anti-inflammatory, antioxidant, and antiapoptotic effects and counteract MSG-induced neurotoxicity by altering the NF-*κ*B and p38 MAPK signaling pathways.

## 1. Introduction

Food additives have serious effects on the brain's functional capabilities [1]. Monosodium glutamate (MSG) is one of the most widely used food additives and has been reported in experiments on animals and people to have significant side effects, including functional, learning, and behavioral impairments [2]. Glutamate, an essential component of MSG, is a nonessential amino acid and is the most important excitatory neurotransmitter in the human brain [1]. It can have either physiological or pathological effects depending on its levels and the susceptibility of a particular brain region [3]. Studies have also shown that MSG-induced obesity is associated with gender as well as aging [4]. The hippocampus has a larger density of glutamate receptors than other brain regions, making it one of the regions most vulnerable to excitotoxic injury [5].

Recent studies have found that MSG induces neurotoxicity through two pathways: it affects the oxidative stress balance within the antioxidant system and it affects the cholinergic system in which acetylcholinesterase (AChE) plays a significant role [6]. An essential mechanism in the control of cell proliferation and apoptosis is the signal transduction of p38 mitogen-activated protein kinase (p38 MAPK) [7]. In neonatal rats, MSG-induced changes in the expression level of the MAPK pathway led to neuronal apoptosis in the hippocampus [5]. On the other hand, the occurrence of neurotoxic and neurodegenerative diseases is significantly influenced by cytokine overabundance in the brain [3]. It has been demonstrated that elevated TNF-*α* induced tissue damage through an inflammatory mechanism associated with the activation of nuclear factor-B (NF-B) [8].

Currently, there is no effective treatment for neurodegenerative diseases. The treatments given to patients remedy the symptoms or slow the disease's progression, but the root causes are left untreated [6]. There is still a critical need to develop new treatments that can counter or even stop the diseases. Recently, a number of dietary phospholipids have been found to be effective at scavenging free radicals, prompting hope that they could be beneficial for neurodegenerative diseases by reducing reactive oxygen species (ROS) [9].

Plasmalogens (Pls) are naturally occurring glycerophospholipids that are primarily located in cell membranes [10]. Pls are characterized by a glycerol backbone that has a vinyl ether bond at the 1-position and an ester bond at the 2-position [11]. Pls are present in almost all mammalian tissues and comprise ~18% of all cell membrane's phospholipid content [10]. Over the last 20 years, there has been a growing interest in Pls due to their roles in bodily functioning and their association with a variety of diseases. Various authors have linked the action of Pls with beneficial mechanisms, primarily through in *in vitro* studies [9]. The identified mechanisms have included reducing inflammatory responses, preventing oxidative stress, and assisting in the maintenance of cell membrane properties with implications for membrane fusion and signal transduction processes like cholesterol efflux [11]. Given the neurodegenerative effects of MSG exposure and the promising neuroprotective potential of Pls, this study was designed to assess the effects of Pls on glutamate-induced neurotoxicity in the hippocampal tissue of male rats, with a focus on how it might suppress apoptosis, reduce oxidative stress and inflammation, and alter p38 MAPK signaling molecules.

## 2. Materials and Methods

### 2.1. Chemicals

MSG was obtained from Sigma–Aldrich Chemical Company (St. Louis, MO, USA). The rest of the compounds were of analytical grade.

### 2.2. Plasmalogens

Pls were extracted from scallops via hokkaido scallop oil plasmalogen (HSOP) according to Kadokawa et al. [12]. Briefly, 1 kg of scallops was homogenized in 3.75 L of methanol/chloroform (2:1, v/v) and incubated at room temperature for 30 min. 1 L of chloroform was then mixed with samples, followed by adding 1 L of water, mixing, and centrifuging for 5 min at 1500×g. The lower layer of chloroform was collected in a glass tube, and 1 L of chloroform was used to re-extract the upper layer. The collective chloroform layers were dried (45°C) with N2 gas, following which 40 mL of 0.1 M citric acid buffer (pH 4.5) with 100 mg/mL phospholipase A1 was added to the tube, the tube was capped (with an N2 gas-filled cap), and the sample was incubated for 1 h at 45 °C. The reaction mixture was extracted twice with chloroform and dried.

### 2.3. Preparation of Pls for Characterization

A 10-mg dried Pls was dissolved in 1 mL diethyl ether and 20 mL methyl acetate. Two hundred and fifty milliliters of 1 M sodium methoxide in methanol was added. The sample was worked up and left for 5 min at room temperature. Saturated oxalic acid solution (35 mL) was added, with brief agitation, to neutralize the solution. The solvent was removed under nitrogen, and 1 mL of hexane was added.

### 2.4. HPLC Analysis of Pls

High-performance liquid chromatography (HPLC) analyses were performed as described by Murphy et al. [13] an Agilent HPLC system (HP1200 Series, Agilent Technologies, Santa Clara, California, USA) equipped with an evaporative light scattering detector (ELSD), a fluorescence detector and a UV detector. Solvent A was a mixture of Hexane/2-propanol/acetic acid (82:17:1 v/v/v) + 0.08% triethylamine, and B was a mixture of 2-propanol/water/acetic acid (85:14:1 v/v/v) + 0.08% triethylamine. Pls were eluted with a gradient of solvent B increasing from 5% to 30% in 20 min, and from 30% to 85% in 3 min, steady at 85% for 2 min and decreasing again from 85% to 5% in 10 min at a flow rate of 0.8 mL/min. The identification of Pls was performed using a standard reagent for ethanolamine-type plasmalogen (PlsEtn).

### 2.5. GC–MS Analysis of Pls

GC–MS analysis of Pls was carried out according to Hanuš et al. [14] using the Perkin Elmer Clarus 600/560 D gas chromatograph (USA) equipped with a 5971B mass selective detector. Pls were analyzed by GC–MS using an RTX-l capillary column: length, 60 m; internal diameter, 0.32 mm; and film thickness, 0.25 mm (Restek, Bellefonte, PA). The GC oven program had an initial temperature of 40°C for 2 min, a 2°C/min run to 300°C, and a final hold at 300°C (20 min). The injector temperature was kept at 180°C (splitless), and the carrier gas (helium) flow rate was 25 cm/s. The MS detector was operated at 194°C, and the scan range was from 30 to 650 m/z at 0.9 scan/set scan rate. The solvent delay was 3 min. Interpretation on mass spectrum was conducted using the database of National Institute Standard and Technology (NIST).

### 2.6. Animals

Twenty-four adult male Wistar albino rats weighing 160–180 g were obtained from the Experimental Animal Center, Medical Research Institute, Alexandria University (Alexandria, Egypt). They were housed in standard stainless steel wire cages and given food and water ad libitum. The animal house was maintained on a 12 h light–dark cycle and kept constantly at 25°C. All experimental procedures were performed according to the guidelines of the Alexandria University Egypt Institutional Animal Care and Use Committee (ALEXU-IACUC approval number: AU 04 23 03 28 3 01).

### 2.7. Experimental Procedure

The rats were randomly assigned to one of four groups, with each group consisting of six rats. Rats in each group were given treatment for 28 days. The treatments for each group were as follows:

• Group 1 (Control): 0.9% saline (gavage)
• Group 2 (MSG): MSG (2 g/kg-body-weight, gavage)
• Group 3 (MSG + Pls): MSG (2 g/kg-body-weight, gavage) + Plasmalogen (100 mg/kg-body-weight, gavage)
• Group 4 (Pls): Plasmalogen (100 mg/kg-body-weight, gavage)

The doses of MSG and Pls were based on experiments by Newairy et al. [15] and Smith et al. [9], respectively.

### 2.8. Classic Labyrinth Test for Neurobehavioral Evaluation

One simple method for evaluating rodent behaviors such as anxiety, memory, or learning ability is to conduct the Classic Labyrinth Test. Our protocol was based on the protocol in the study of Gasmi [16]. In short, we used a square-shaped maze with locations for starting and stopping. Once animals are trained, they can be given 10 min to freely look at and explore the maze. The animal's horizontal and vertical movements within the maze are recorded during this time interval. The animal must remember the fastest path from start to finish, making this a potentially difficult task. If the maze is designed so animals only need to walk, the task can be fairly easy for healthy rats. For rats suffering from the effects of neuro-xenobiotics such as drugs or pesticides, the rats will exhibit disturbances on their journey through the labyrinth. For controlled trials, 10% of ethanol was used to clean the labyrinth after every rat pass.

### 2.9. Biochemical Estimation

A commercial diagnostic kit (Biodiagnostics, Egypt) was used to measure serum glucose levels. Sandwich ELISA was used to measure serum insulin levels using kits purchased from Linco Research, USA. A commercial kit (Sigma, USA) was used to measure serum total cholesterol (TC) levels. A kit from Sigma Chemical Co. was used to enzymatically measure the serum triglyceride (TG) concentration. The concentrations of malondialdehyde (MDA) and glutathione (GSH) in the hippocampus homogenate were measured according to methods detailed by Ohkawa et al. [17] and Ellman [18], respectively. Commercial kits (Bio Systems S.A.) were used to calorimetrically measure nitric oxide (NO) levels. The activities of glutathione peroxidase (GPx; EC: 1.11.1.9) and superoxide dismutase (SOD; EC: 1.15.1.1) were measured in hippocampus homogenate using the methods of Rotruck et al. [19] and Nishikimi et al. [20], respectively. ELISA kits (MyBioSource, San Diego, USA) were used to measure the levels of serotonin (5-HT, Catalog # MBS702675), gamma-hydroxybutyric acid (GABA, Catalog # MBS740443), acetylcholine (ACh, Catalog # MBS728879), and AChE (EC 3.1.1.7, Catalog # MBS038896). Rat immunoassay kits (MyBioSource, San Diego, USA) were used to measure TNF-*α* (Catalog # MBS175904), IL-1*β* (Catalog # MBS2023030), and amyloid *β*_1-42_ (Catalog # MBS2023749) by ELISA. Phospho-AKT was measured by ELISA (CAT. No. K4211-100) obtained from Biovision.

### 2.10. Western Blot Analysis

Hippocampus tissues were homogenized in radioimmunoprecipitation (RIPA) buffer containing protease inhibitors. Bicinchoninic acid (BCA) assay kit (Pierce, USA) was used to measure the protein concentration. SDS polyacrylamide gel was then used to separate protein (50 μg/well) for transfer to a polyvinylidene fluoride (PVDF) membrane (Millipore, USA). This was subsequently blocked with Tris-buffered saline containing 5% BSA and 0.05% Tween 20. Rabbit monoclonal p38MAPK (dilution 1:1000, Cell Signalling Technology), Rabbit monoclonal NF-*κ*B (dilution 1:1000, Cell Signalling Technology), and mouse monoclonal anti-*β*-actin (1:2000, Sigma–Aldrich) were diluted in TBST according to manufactured instructions. Incubation was done overnight for each primary antibody solution against the blotted target protein, at 4°C. The blot was rinsed 3–5 times for 5 min with TBST. Incubation was done in the HRP-conjugated secondary antibody (Goat antirabbit IgG-HRP-1 mg Goat mab Novus Biologicals, dilution 1:2000) solution against the blotted target protein for 1 h at the room temperature. The blot was rinsed 3–5 times for 5 min with TBST. Chemiluminescence reagent (Millipore, USA) was used to visualize protein bands. A *β*-actin antibody was used to confirm equivalent loading, and protein band levels of the target proteins were measured densitometrically with Image J. Three independent experiments were used for selecting representative blots [21].

### 2.11. Histological and Immunohistological Assessments

Hippocampus tissues were fixed in 10% neutral buffered formalin solution, after which they were dehydrated, cleared, and embedded in paraffin wax. 5 μm sections were taken, stained with hematoxylin and eosin (H&E), and microscopically examined for evidence of histopathological changes [22]. Quantitative analysis was conducted on histological sections obtained from three animals per experimental group, with two slides analyzed per animal. For immunohistological assessment, the 5-μm-thick paraffin sections were deparaffinized for 1–2 min in xylene and then rehydrated with ethanol of descending grades (100%, 95%, and 70%), each for 5 min. They were then combined for another 5 min with distilled water. PBS was then used to rinse the sections, and they were blocked with 0.1% H_2_O_2_ for 30 min to inhibit endogenous peroxidase activity. PBS was again used for rinsing, and the sections were incubated in blocking solution (10% normal goat serum) at room temperature (RT, 21°C) for 60 min, and then incubated for another hour at RT with the primary antibody (Synaptophysin Catalog #MBS9600194 dilution 1:100 and iNOS Catalog # MBS9600137 dilution 1:100). PBS was again used for rinsing, sections were incubated at RT for 20 min with the secondary biotinylated antibody, they were rinsed again with PBS, and enzyme conjugate “Streptavidin-Horseradish peroxidase” solution was applied for 10 min to the sections. A solution of 3,3′-diaminobenzoic acid (DAB) dissolved in PBS was used to visualize secondary antibody binding, with H_2_O_2_ being added to the solution immediately before use to a concentration of 0.03%. Finally, PBS was used to rinse the sections, and the slides were counterstained with two drops of 100 µL of hemotoxylin. Distilled water was used to wash the slides until the sections turned blue. Slides were then dehydrated in ethanol of ascending grades (70%, 95%, and 100%) for 5 min each, cleared in xylene, and covered using histomount mounting solution. Six independent experiments were conducted. For the quantitative assessment of immunohistology, photographs of the hippocampus were randomly captured with a digital camera (Olympus) of five nonoverlapping fields (400×) per section; the whole dentate gyral area was analyzed for each hippocampal section for each marker [23].

### 2.12. Statistical Analysis

Data are shown as mean ± standard error for each animal. Statistical significance was assessed with the one-way analysis of variance (ANOVA) and Tukey's post hoc multiple comparison tests. Results are reported to be statistically significant for *p*  < 0.05.

## 3. Results

### 3.1. Pls Characterization

**Figure 1:**
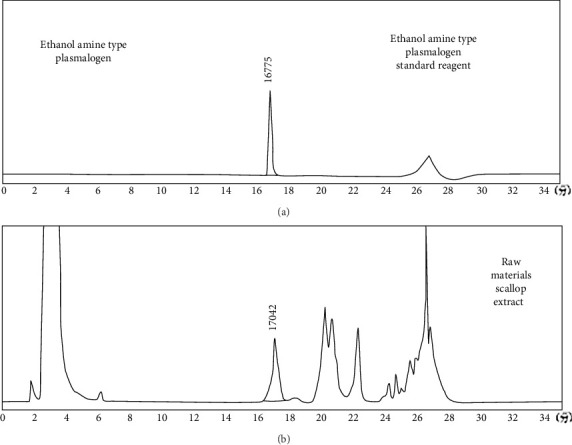
HPLC chromatogram of (A) ethanolamine-type plasmalogen standard and (B) Pls extracted from scallops.

**Figure 2:**
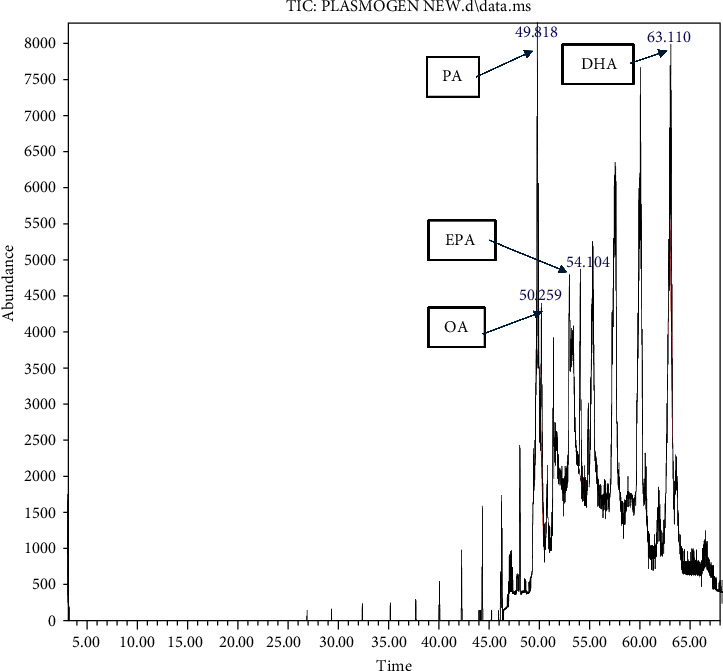
GC–MS chromatogram of Pls showing palmitic acid (PA) 16:0, oleic acid (OA) - 18:1(n-9), eicosapentaenoic acid (EPA) - 20:5(n-3) and docosahexaenoic acid (DHA) - 22:6(n-3).

HPLC analysis (Figure 1) revealed the composition of scallop-derived Pls is 97% PlsEtn and other glycerophospholipids. While GC–MS analysis of Pls (Figure 2) showed palmitic acid (PA) 16:0, oleic acid (OA) 18:1(n-9), eicosapentaenoic acid (EPA) 20:5(n-3) and docosahexaenoic acid (DHA) 22:6(n-3).

### 3.2. Classic Labyrinth Test for Neurobehavioral Evaluation

MSG-treated rats spent a significantly (*p*  < 0.05) longer amount of time in the maze compared to control. The time decreased significantly (*p*  < 0.05) with the administration of Pls (Table 1).

### 3.3. Serum Levels of Glucose, Insulin, and Lipids

Results in Table 2 show a remarkable increase in serum glucose, cholesterol, and TGs with a decline in serum insulin in the MSG-treated group, while supplementation with Pls ameliorates these parameters.

### 3.4. Levels of Antioxidant Enzymes and Hippocampal Oxidative Stress Markers

MDA and NO levels markedly increased for MSG-treated rats compared to the control, while the GSH levels and SOD and GPx activities decreased (Table 3). In contrast, treatment with MSG plus Pls showed a decline in the levels of MDA and NO, as well as elevated GSH levels and SOD and GPx activities in the hippocampus compared to the MSG-treated group.

### 3.5. Neurochemical Estimations

Serotonin and ACh concentrations showed a marked reduction in the group treated with MSG relative to the control. These concentrations improved with Pls treatment. Moreover, GABA and AChE levels revealed a marked elevation in the group treated with MSG relative to the control. Pls treatment ameliorated this elevation as well (Table 4).

### 3.6. Inflammatory Markers

The results in Table 5 show a remarkable elevation in hippocampal TNF-*α*, IL-1*β*, and amyloid *β*_1-42_ and a reduction in AKT in the MSG-treated group. Their concentrations improved with Pls treatment to reach nearly normal values.

### 3.7. NF-κB and p38 MAPK Expression

**Figure 3:**
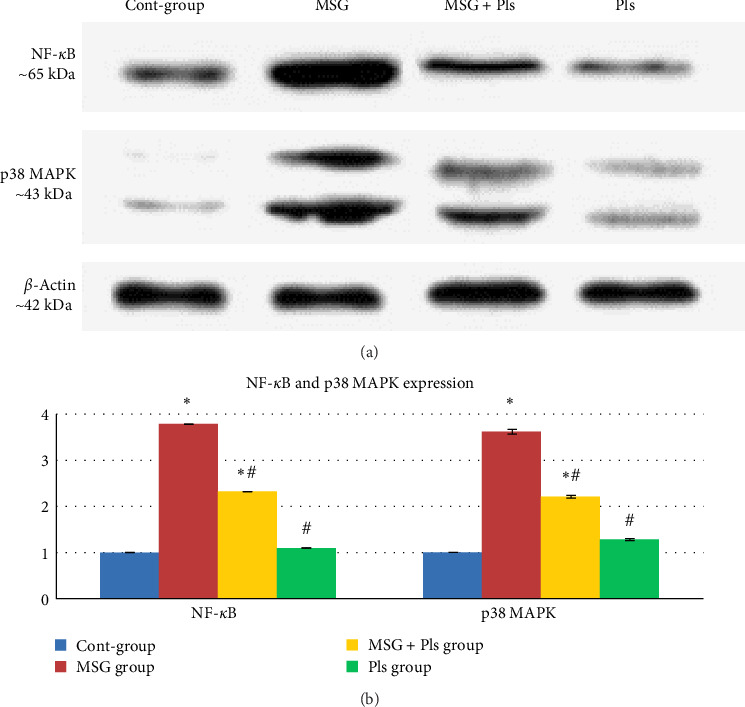
Western blot analysis showing the effect of plasmalogen (Pls) on protein levels of NF-*κ*B and MAPK in the hippocampal tissues of rats exposed to monosodium glutamate (MSG). (A) shows representative blots, while (B) shows mean values over all blots normalized relative to the control. *⁣*^*∗*^ Significantly different from control group at *p*  < 0.05, ^#^ Significantly different from MSG group at *p*  < 0.05.

Results in Figure 3 show upregulation of NF-*κ*B and p38 MAPK protein levels for the MSG-treated group. On the other hand, their concentrations were significantly enhanced post exposure to Pls.

### 3.8. Histological and Immunohistological Assessments

**Figure 4:**
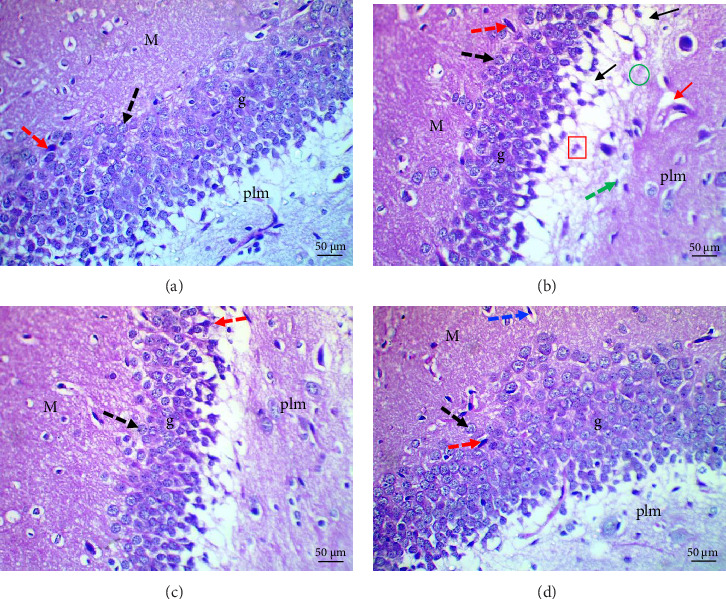
Photomicrographs of sections of dentate gyrus of control (A) and Pls-treated (D) rats showing normal molecular layer (M) with normal blood vessel (blue dotted arrow), granular layer (g) with normal granular cells (black dotted arrow), normal pyramidal cells (red dotted arrow) and normal polymorphic (plm) layer. MSG-treated rats (B) show neurodegenerated cells (red square), pericellular vacuole (black arrow), pyknotic nuclei (green dotted arrow), dilated blood vessel (red arrow) and encephalomalacia (green circle). A marked improvement is seen in MSG + Pls-treated rats (C) (H&E X400).

Histological evaluation of the dentate gyrus of control and Pls-treated rats showed normal molecular layers with normal blood vessels, normal granular layers with normal granular cells, normal pyramidal cells, and normal polymorphic layers (Figure 4A,D). A similar evaluation of the MSG-treated group found neuronal degeneration associated with apoptotic granular cells and the presence of cellular vacuolation and encephalomalacia (Figure 4B). In contrast, administration of Pls plus MSG resulted in neurons that looked visibly proximal to the control (Figure 4C). MSG exposure significantly increases neuronal damage in the hippocampus of male rats. However, the addition of Pls to the MSG treatment quantitatively mitigated the neuronal damage caused by MSG (Table 6), suggesting a neuroprotective effect of Pls. Notably, when administered independently, Pls did not have any significant effect on neuronal integrity.

**Figure 5:**
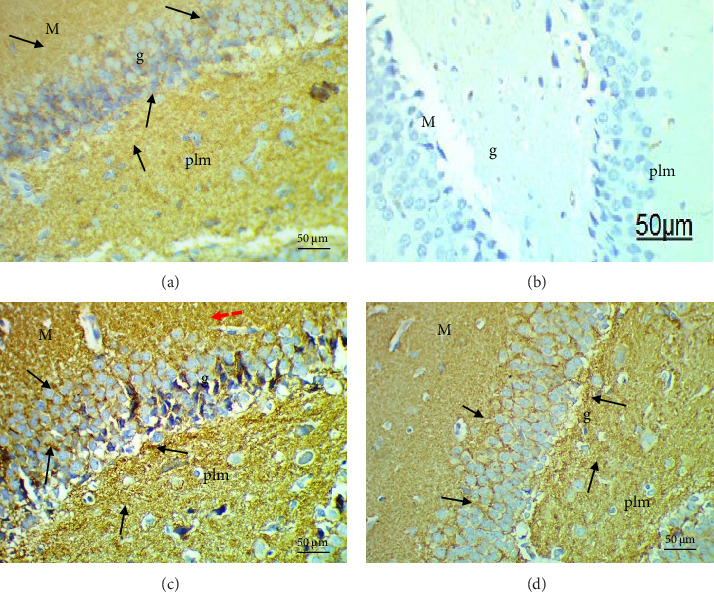
Photomicrographs of sections in rat's hippocampus showing immunoreactivity for synaptophysin (CYV). Control and Pls-treated rats (A, D) show positive reaction for CYV (black arrow). In contrast, the hippocampus section from the MSG-treated group (B) shows weak reaction for CYV, while that of the MSG + Pls-treated group (C) shows positive reaction for CYV (black arrow) (CYV, X400).

**Figure 6:**
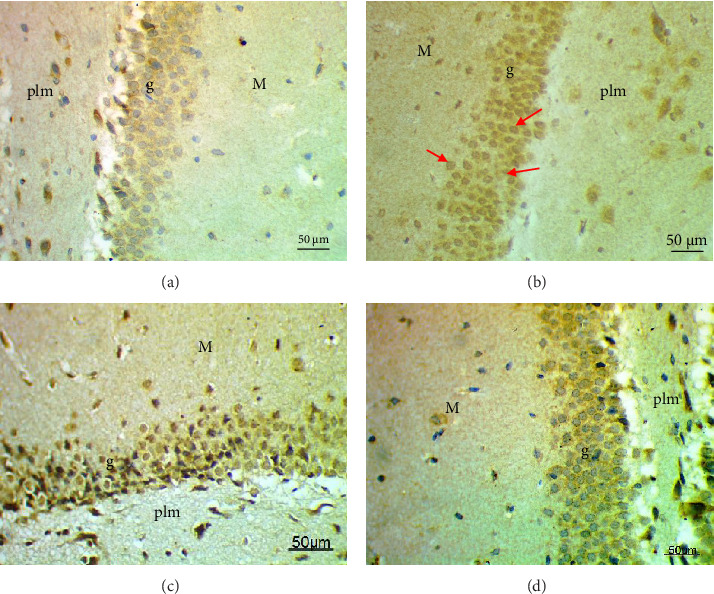
Photomicrographs of sections in rat's hippocampus showing immunoreactivity for iNOS. Sections of the control and Pls-treated groups (A, D) show weak immunostaining for iNOS. The hippocampus section from the MSG-treated group (B) shows strong reaction for iNOS (red arrow), while that of the MSG + Pls-treated group (C) shows slightly weak immunostaining for iNOS (iNOS, X400).

The cell proliferation marker iNOS and the major synaptic vesicle protein synaptophysin were used to assess the proliferating cell count (Figures 5 and 6; Table 7). Sections in control and Pls-treated rats showed positive reactions for synaptophysin (Figure 5A,D). There was a weak reaction for synaptophysin in the group treated with MSG (Figure 5B). In contrast, there was a more positive reaction for synaptophysin for rats treated with Pls plus MSG relative to those treated only with MSG (Figure 5C). Furthermore, weak iNOS immunostaining was observed in the cytoplasm of neurons in the hippocampi of control and Pls-treated rats (Figure 6A,D). The hippocampal sections of rats treated with MSG showed a strong reaction for iNOS (Figure 6B). Coadministration of MSG + Pls showed slightly weak immunostaining for iNOS in the hippocampal neurons (Figure 6C).

## 4. Discussion

One of the most frequently used food additives is MSG [24], despite the fact that it has neurodegenerative effects. Results of this study revealed that scallop-derived Pls exert a potent protective effect against MSG via different signaling pathways in the hippocampus using behavioral, biochemical, and immunohistochemical methods. The present data show notable variations in behavioral performance between the control group and rats treated with MSG. MSG exerts excitotoxic effects on the brain, leading to significant impairment in short-term memory and alterations in exploratory behavior in rats [25]. These excitotoxic impacts of MSG come from glutamate interaction with its receptors, promoting apoptosis and necrosis of neuronal cells [6]. Conversely, Pls demonstrated efficacy in mitigating the memory impairments observed in animals exposed to MSG. Studies suggest that Pls might improve memory function and reduce the generation of pro-inflammatory cytokines in the cortex and hippocampus of mouse brains [26].

We also found that rats treated with MSG had significantly enhanced glucose, cholesterol, and TG levels and a decline in serum insulin. This agrees well with earlier studies [27, 28]. Elevated glucose levels have been strongly linked to increased oxidative stress and inflammation, both of which are recognized contributors to neurodegenerative processes [29]. Hyperglycemia stimulates the generation of ROS through multiple pathways, including glucose autoxidation, the activation of the polyol pathway, and the overproduction of advanced glycation end products [30]. The accumulation of ROS overwhelms the antioxidant defense systems, resulting in oxidative damage to cellular macromolecules such as lipids, proteins, and DNA, which is a hallmark of neurodegenerative conditions similar to Alzheimer's disease [31]. Additionally, elevated glucose levels activate inflammatory signaling pathways leading to the release of pro-inflammatory cytokines [29]. Chronic inflammation induced by these cytokines contributes to neuronal dysfunction and cell death, further exacerbating neurodegenerative processes [32]. Similarly, high cholesterol disrupts neuronal membranes and increases amyloid-beta aggregation, while elevated TGs promote oxidative stress and systemic inflammation [33]. These findings emphasize the critical role of glucose and lipid metabolism in maintaining neuronal health and highlight the pathological consequences of MSG in neurodegenerative diseases. Treatment with Pls reduced the elevated levels of glucose, cholesterol, and TGs and improved serum insulin. Dietary phospholipids have been reported to reduce plasma cholesterol, perhaps as a result of their ability to restrict gastrointestinal absorption of cholesterol [10].

The brain is very susceptible to oxidative stress damage, due to its high oxidative metabolic activity, high concentration of polyunsaturated fatty acids, abundance of redox-active transition metal ions, moderately low antioxidant capacity, and the fact that it is composed of neuronal cells that are nonreplicating in nature [6]. In this study, the increase in hippocampal MDA and NO levels are associated with decreases in the GSH level and in the SOD and GPx activities. This is in accordance with recent investigations [15]. However, it was of great interest to note that administration with Pls led to significantly ameliorated levels of oxidative stress parameters. Pls ability to act as a potent antioxidant agent could be due to the enhancement of electron density of the vinyl ether bond at the 1-position that leaves it prone to cleavage by ROS, or to the enhancement at the vinyl ether linkage position, which has been posited to be in the membrane's hydrophilic domain and therefore prone to ROS attack [11].

We also found that MSG inhibited serotonin and ACh concentrations and augmented GABA and AChE levels. Previous findings have also demonstrated a decline in Ach in the synaptic cleft due to MSG, leading to cellular degeneration and an increased concentration of cholinesterase in brain tissues [15]. The current data show that daily intake of MSG plus Pls leads to recovery of brain levels of serotonin, GABA, ACh, and AChE activity. This is in accordance with other work [34]. Considering that Pls are key lipid components facilitating membrane fusion of synaptic vesicles involved in the release of neurotransmitters, changes in Pls content are thought to play essential roles in neurological disorders [35].

Previous work has shown that MSG exposure causes an increase in hippocampal TNF-*α*, IL-1*β*, and A*β* and a reduction in AKT. The underlying mechanism may involve its ability to boost pro-inflammatory cytokine production for cytokines that specifically bind to the promoter region of the gene that NF-*κ*B targets to start an inflammatory response [8]. On the other hand, Pls inhibit this process. Pls could lower the amount of pro-inflammatory cytokines and diminish the burden of A*β* accumulation, both of which are related to the expression of cytokines in the brain [26]. Further study has shown that Pls prevent the PI3K-AKT/mTOR and MAPK signaling pathways from being activated [36].

The MSG-treated group of this study exhibited upregulation of NF-*κ*B and p38 MAPK protein levels. The increase in extracellular glutamate concentration can activate the p38 MAPK signaling pathway, a pathway implicated in various apoptotic processes [7]. Our results further demonstrated that combining MSG with Pls inhibited the induction of NF-*κ*B and p38 MAPK proteins in the hippocampus. Research indicated that PUFA-containing Pls can reduce NO generation in glial cells through inhibition of the NF-*κ*B, JNK, and p38 MAPK pathways [37]. Unlike the previous study, the p38 MAPK was unchanged despite in our study it was decreased. The detailed inhibitory mechanism of Pls on p38 MAPK remains uncharacterized in this investigation. We suggest that it might affect the gene expression of p38 MAPK thus leading to a decrease in the protein level. Further research on the gene level is needed to prove this suggestion.

Our histopathological study shows neuronal structural loss and necrosis in the hippocampi of rats treated with MSG, which corroborates with other's findings [38, 39]. The observed effects may be attributed to MSG's induction of oxidative damage [40]. Pls ameliorated these negative effects and restored hippocampal tissues without histopathological alterations, suggesting Pls's protective effect against excitotoxic neuronal damage induced by MSG in hippocampi. This could be a result of Pls role in scavenging free radicals [11]. Moreover, the administration of Pls could improve hippocampal neurogenesis and suppress hippocampal-dependent behavioral deficits in AD mice [41].

The present study demonstrated significant improvements in synaptophysin expression following Pls administration to rats given MSG; this finding may be attributed to Pls ability to prevent the degenerative alterations of synapses caused by MSG. This is in accordance with recent studies demonstrating that Pls are associated with neurogenesis in the hippocampus, anti-inflammatory effects, and increased synaptic plasticity [42]. The current study and others' work show that MSG treatment can activate NF-*κ*B, resulting in overexpression of its target genes such as IL-6, iNOS, and TNF-*α* which contributes to cell damage [8, 15]. Importantly, rats treated with MSG plus Pls had weak iNOS immunostaining, suggesting that Pls protective action is partly dependent on its antioxidative properties [11]. The depletion of Pls might contribute to synaptic dysfunction. These crucial lipids are essential for facilitating membrane fusion within synaptic vesicles, a process directly linked to neurotransmitter release [41]. Conversely, the administration of Pls has been demonstrated to inhibit the production of A*β* by regulating *γ*-secretase activity and preventing neuronal cell death due to its antiapoptotic properties [26].

Given the presence of DHA and EPA, particularly within the PlsEtn used in this study, the possibility that PlsEtn contributes significantly to the observed neuroprotective effects of Pls.

Prior research has established the neuroprotective effects of Pls due to their antioxidant and anti-inflammatory properties [43]. Evidence suggests that these benefits may be partially attributed to the crucial fatty acids carried at the sn-2 position [41]. DHA and EPA are the primary fatty acids found in this location [44]. These molecules have been shown to inhibit *β*-secretase activity while enhancing *α*-secretase activity, promoting a nonamyloidogenic processing pathway for the amyloid precursor protein (APP) instead of the amyloidogenic pathway [41]. Furthermore, DHA and EPA can improve the degradation and clearance of A*β*, ultimately resulting in a reduction of A*β* levels and amyloid fibril formation [41, 43].

## 5. Conclusion

The present investigations demonstrate that scallop-derived Pls possess a neuroprotective role against MSG-induced neurodegeneration in rats. The underlying mechanisms may include the ability of Pls to cause ROS scavenging, reduce oxidative stress, exert anti-inflammatory and antiapoptotic effects, and ultimately prevent the disruption of the NF-*κ*B and p38 MAPK signaling pathways. These suggest that Pls may have beneficial therapeutic applications. Further studies are needed to validate Pls role in the improvement of brain injury.

## Figures and Tables

**Table 1:** Classic labyrinth test for neurobehavioral evaluation.

Parameters	Experimental groups			
	Control group	MSG	MSG + Pls	Pls
Memory-related behavioral tests (sec)	98.00 ± 6.81	193.33 ± 8.82*⁣*^*∗*^	145.67 ± 3.48*⁣*^*∗*^^#^	95.00 ± 2.89^#^

*Note:* Values are expressed as means ± S.E.; *n* = 6 for each group.

Abbreviation: MSG, monosodium glutamate.

*⁣* ^*∗*^The mean values are significantly different compared to the control group at *p*  < 0.05.

^#^The mean values are significantly different compared to the MSG group at *p*  < 0.05.

**Table 2:** Serum levels of glucose, insulin and lipid profile.

Parameters	Experimental groups			
	Control group	MSG	MSG + Pls	Pls
Glucose (mg/dl)	95.17 ± 3.07	274.50 ± 5.81*⁣*^*∗*^	155.67 ± 1.58*⁣*^*∗*^^#^	83.00 ± 3.22*⁣*^*∗*^^#^
Insulin (mIU/L)	2.03 ± 0.03	0.98 ± 0.02*⁣*^*∗*^	1.43 ± 0.04*⁣*^*∗*^^#^	1.93 ± 0.03*⁣*^*∗*^^#^
Cholesterol (mg/dl)	67.17 ± 1.81	165.00 ± 4.86*⁣*^*∗*^	62.33 ± 4.24^#^	52.50 ± 1.67*⁣*^*∗*^^#^
Triglyceride (mg/dl)	48.50 ± 1.73	95.33 ± 2.39*⁣*^*∗*^	60.83 ± 4.21*⁣*^*∗*^^#^	47.00 ± 1.32^#^

*Note:* Values are expressed as means ± S.E.; *n* = 6 for each group.

Abbreviation: MSG, monosodium glutamate.

*⁣* ^*∗*^The mean values are significantly different compared to the control group at *p*  < 0.05.

^#^The mean values are significantly different compared to the MSG group at *p*  < 0.05.

**Table 3:** Levels of brain oxidative stress markers and antioxidant enzymes.

Parameters	Experimental groups			
	Cont-group	MSG	MSG + Pls	Pls
MDA (nmole/g tissue)	3.22 ± 0.14	10.83 ± 0.09*⁣*^*∗*^	5.87 ± 0.11*⁣*^*∗*^^#^	4.08 ± 0.15^#^
NO (µmol/g tissue)	6.77 ± 0.20	18.42 ± 0.97*⁣*^*∗*^	9.77 ± 0.32*⁣*^*∗*^^#^	6.97 ± 0.16^#^
GSH (nmol/g tissue)	48.67 ± 0.88	27.50 ± 1.26*⁣*^*∗*^	41.50 ± 0.76*⁣*^*∗*^^#^	49.33 ± 1.52^#^
SOD (U/g tissue)	8.97 ± 0.21	3.50 ± 0.29*⁣*^*∗*^	5.80 ± 0.22*⁣*^*∗*^^#^	8.93 ± 0.33^#^
GPX (U/g tissue)	11.08 ± 0.31	4.85 ± 0.28*⁣*^*∗*^	8.80 ± 0.20*⁣*^*∗*^^#^	11.80 ± 0.35^#^

*Note:* Values are expressed as means ± S.E.; *n* = 6 for each group.

Abbreviation: MSG, monosodium glutamate.

*⁣* ^*∗*^The mean values are significantly different compared to the control group at *p*  < 0.05.

^#^The mean values are significantly different compared to the MSG group at *p*  < 0.05.

**Table 4:** Neurochemical estimations.

Parameters	Experimental groups			
	Control group	MSG	MSG + Pls	Pls
Serotonin (ng/g tissue)	36.50 ± 0.76	13.67 ± 0.88*⁣*^*∗*^	29.50 ± 0.62*⁣*^*∗*^^#^	37.00 ± 1.13^#^
GABA (pg/g tissue)	20.37 ± 0.74	39.90 ± 0.76*⁣*^*∗*^	28.18 ± 0.53*⁣*^*∗*^^#^	21.05 ± 0.60^#^
ACh (U/g tissue)	15.00 ± 0.16	7.47 ± 0.31*⁣*^*∗*^	12.83 ± 0.17*⁣*^*∗*^^#^	13.97 ± 0.15*⁣*^*∗*^^#^
AChE (pg/g tissue)	9.55 ± 0.21	20.40 ± 0.37*⁣*^*∗*^	14.92 ± 0.39*⁣*^*∗*^^#^	11.43 ± 0.25*⁣*^*∗*^^#^

*Note:* Values are expressed as means ± S.E.; *n* = 6 for each group.

Abbreviation: MSG, monosodium glutamate.

*⁣* ^*∗*^The mean values are significantly different compared to the control group at *p*  < 0.05.

^#^The mean values are significantly different compared to the MSG group at *p*  < 0.05.

**Table 5:** Inflammatory markers.

Parameters	Experimental groups			
	Control group	MSG	MSG + Pls	Pls
TNF-*α* (pg/g tissue)	31.83 ± 0.79	72.50 ± 1.65*⁣*^*∗*^	47.67 ± 2.32*⁣*^*∗*^^#^	30.67 ± 0.67^#^
IL-1*β* (pg/g tissue)	7.35 ± 0.10	19.03 ± 0.71*⁣*^*∗*^	13.03 ± 0.27*⁣*^*∗*^^#^	7.03 ± 0.32^#^
Amyloid *β*_1-42_ (pg/g tissue)	14.37 ± 0.17	28.17 ± 1.47*⁣*^*∗*^	18.67 ± 0.40*⁣*^*∗*^^#^	14.80 ± 0.24^#^
AKT (µg/g tissue)	2.65 ± 0.09	1.08 ± 0.11*⁣*^*∗*^	1.80 ± 0.07*⁣*^*∗*^^#^	2.66 ± 0.11^#^

*Note:* Values are expressed as means ± S.E.; *n* = 6 for each group.

Abbreviation: MSG, monosodium glutamate.

*⁣* ^*∗*^The mean values are significantly different compared to the control group at *p*  < 0.05.

^#^The mean values are significantly different compared to the MSG group at *p*  < 0.05.

**Table 6:** Quantification of lesions detected in histological sections of hippocampus tissue stained with H&E.

Parameters (Lesion Count)	Experimental groups			
	Control group	MSG	MSG + Pls	Pls
Neurodegenerated cells	2.17 ± 0.31	18.83 ± 0.60*⁣*^*∗*^	8.50 ± 0.62*⁣*^*∗*^^#^	2.00 ± 0.26^#^
Cells with pyknotic nuclei	1.33 ± 0.21	8.17 ± 0.31*⁣*^*∗*^	4.33 ± 0.49*⁣*^*∗*^^#^	1.17 ± 0.17^#^
Pericellular vacuole	5.17 ± 0.31	39.33 ± 1.41*⁣*^*∗*^	7.67 ± 0.71^#^	5.50 ± 0.50^#^
Encephalomalacia	1.83 ± 0.31	12.00 ± 0.93*⁣*^*∗*^	2.67 ± 0.21^#^	1.50 ± 0.22^#^
Dilated blood vessel	1.50 ± 0.34	4.67 ± 0.33*⁣*^*∗*^	2.00 ± 0.37^#^	1.33 ± 0.21^#^

*Note:* Values are expressed as means ± S.E.; *n* = 6 for each group.

Abbreviation: MSG, monosodium glutamate.

*⁣* ^*∗*^The mean values are significantly different compared to the control group at *p*  < 0.05.

^#^The mean values are significantly different compared to the MSG group at *p*  < 0.05.

**Table 7:** Changes in the mean number of synaptophysin and iNOS positive cells in the hippocampus of male rats in the different experimental groups.

Parameters (Number of positive cells)	Experimental groups			
	Control group	MSG	MSG + Pls	Pls
Synaptophysin	18.00 ± 1.18	4.17 ± 0.47*⁣*^*∗*^	10.38 ± 0.70*⁣*^*∗*^^#^	19.5 ± 1.05^#^
iNOS	6.50 ± 0.76	16.67 ± 1.02*⁣*^*∗*^	11.50 ± 0.67*⁣*^*∗*^^#^	6.33 ± 0.49^#^

*Note:* Values are expressed as means ± S.E.; *n* = 6 for each group.

Abbreviation: MSG, monosodium glutamate.

*⁣* ^*∗*^The mean values are significantly different compared to the control group at *p*  < 0.05.

^#^The mean values are significantly different compared to the MSG group at *p*  < 0.05.

## Data Availability

The data that support the findings of this study are available from the corresponding author upon reasonable request.
